# Characteristics of the length of the radius and ulna in children

**DOI:** 10.3389/fped.2022.737823

**Published:** 2022-08-09

**Authors:** Chunxing Wu, Dahui Wang, Yueqiang Mo, Zhiqiang Zhang, Bo Ning

**Affiliations:** Department of Pediatric Orthopedics, Children's Hospital of Fudan University, National Children's Medical Center, Shanghai, China

**Keywords:** children, radius, ulna, length, ratio, age, X-ray

## Abstract

**Objectives:**

Congenital malformation, trauma, tumor, or metabolic disease can cause length deformity of the radius or ulna, affecting the appearance and function of the forearm. Osteotomy and lengthening with external fixation can obviously improve the length of the radius and ulna (LRU). However, the extent of lengthening required is still unclear. This study analyzed the LRU in children, to provide suggested standards for various orthopedic treatments.

**Methods:**

Normal LRUs were measured on X-ray images in children who came to hospital for emergency treatment, with measurements including anterior–posterior (AP) radiographs, lateral (LAT) radiographs, full LRU (total length), and LRU without the epiphysis (short length). Any cases of fracture or deformity affecting measurement were excluded. Three hundred twenty-six cases were divided into 16 groups according to age from 1 year old to 16 years old.

**Results:**

The earliest epiphyseal plate and ossification center were observed in the distal part of the radius at 1 year old, and in the proximal part at 3 years old in both boys and girls. In the ulna, at the distal end it was 6 years old in girls and 7 years old in boys, while in the proximal part ossification was observed at 9 years old in both boys and girls. The proximal epiphyseal plate of the ulna began to close on X-ray images at 12 years old in girls and 13 years in boys. LRU increased with age, and there was a strong positive correlation and consistent ratio between radius, ulna and age. In short length, the ratio of the length of radius to ulna (RLRU) ranged from 0.8941 to 0.9251 AP, from 0.8936 to 0.9375 LAT. In total length, RLRU ranged from 0.9286 to 0.9508 AP, and 0.9579 to 0.9698 LAT.

**Conclusions:**

The length and epiphyseal ossification of the radius and ulna are associated with age. RLRU is also limited to a certain range and tends to remain stable with age. These characteristics have clinical significance for deformity correction of the forearm.

## Introduction

Congenital malformation, trauma, tumor, or metabolic disease can cause the radius or ulna to develop too long or too short in some children. Length deformities such as ulnar deviation or radial deviation, cubitus varus or valgus, limited pronation or supination affect the appearance and function of the forearm (1, 2). For deformity caused by differences in the lengths of the ulna and radius, the technique of osteotomy and limb lengthening can be used to correct the deformity by lengthening the short bone (3).

For example, multiple osteochondromas can cause limb deformities. In children with disability due to multiple osteochondromas, lengthening of the radius or ulna can also be used to restore the relationship of the radius to the ulna in the forearm. However, in clinical practice, there is no general agreement regarding the indications for surgery, and the long-term outcomes after surgery remain uncertain (4–10). Consequently, it is difficult to determine what length is considered excessive for limb lengthening. Excessive lengthening may lead to new problems, such as wrist pain, joint impact, or restricted joint function (3, 10, 11). Even if the outcome of the lengthening is as expected, with further growth of the radius or ulna, the affected bone may become relatively shorter again, causing the previous deformity to recur. Accurate knowledge of the expected length can avoid these problems. How to lengthen the affected bone to the ideal length, how to predict the final length and lengthen the affected bone to the target length in advance, and how to achieve optimal recovery with the fewest operations, are all questions which pediatric orthopedic surgeons must consider (12–15).

These treatments require an accurate understanding of the length and angle of the radius and ulna in their natural state. In addition, these demands are increasing. Many researchers have taken physical measurements on adult cadavers, or locally at the elbow, wrist, hand and other landmarks (16, 17). However, there have been few studies on the normal radius and ulna in children (18, 19).

Some studies have been carried out but are mainly based on body surface measurements. For example, Zhang et al. (20) measured the forearm length (body surface) of children aged from 3 to 5 years old, and Chen et al. (21) measured the arm span length or forearm ulnar length in children aged from 1 to 17 years old. Rasouli et al. (22) measured ulnar length using digital calipers to predict body height in children and adults aged 1 month to 23 years, while Edmond et al. (23) measured upper arm length, upper arm circumference, forearm length, and forearm circumference in children aged 0 to 17 years old.

However, the extent of lengthening required is still unclear. Is there a relationship between age and the length of the ulna or radius? Is there a ratio between the length of the ulna and radius? There has been a lack of comprehensive research into these questions. In this study we measured, analyzed and summarized the basic characteristics of the normal length and ratio of the radius and ulna in Chinese children, to provide background data which will help with various orthopedic surgeries.

## Materials and methods

Inclusion criteria: anterior–posterior (AP) and lateral (LAT) radiographs of normal ulna and radius that were measured in the emergency department due to trauma. Patients were divided into 16 groups aged from 1 to 16 years old.

Exclusion criteria: fracture, malformation, neurodevelopmental disorder, and other abnormal images such as tumor, metabolic disease.

Two experienced pediatric orthopedic surgeons received training and measured the dimensions separately on X-rays, and the average of their measurements was taken. Measurements included total length of radius (TLR), total length of ulna (TLU), short length of radius (radius without epiphysis) (SLR), and short length of ulna (ulna without epiphysis) (SLU) on AP and LAT radiographs. The lengths of the midpoint line from the proximal part to the distal part were selected as TLR, SLR, SLU. Because the midpoint of the proximal part of the ulna is difficult to identify both on AP and LAT radiographs, TLU was measured based on the connecting line from the most prominent, nearest point at the proximal part of the ulna to the midpoint of the distal part of the ulna. Data were then analyzed according to age, gender, left/right, AP and LAT, TLR, TLU, SLR, SLU, and ratio of radius to ulna.

SPSS 17 statistical software (SPSS Inc., Chicago, IL, USA) was used for statistical analysis, one-way analysis of variance (ANOVA), reliability analysis, correlation analysis, and regression analysis. *P* < 0.05 indicated a significant difference.

**Figure 1:**
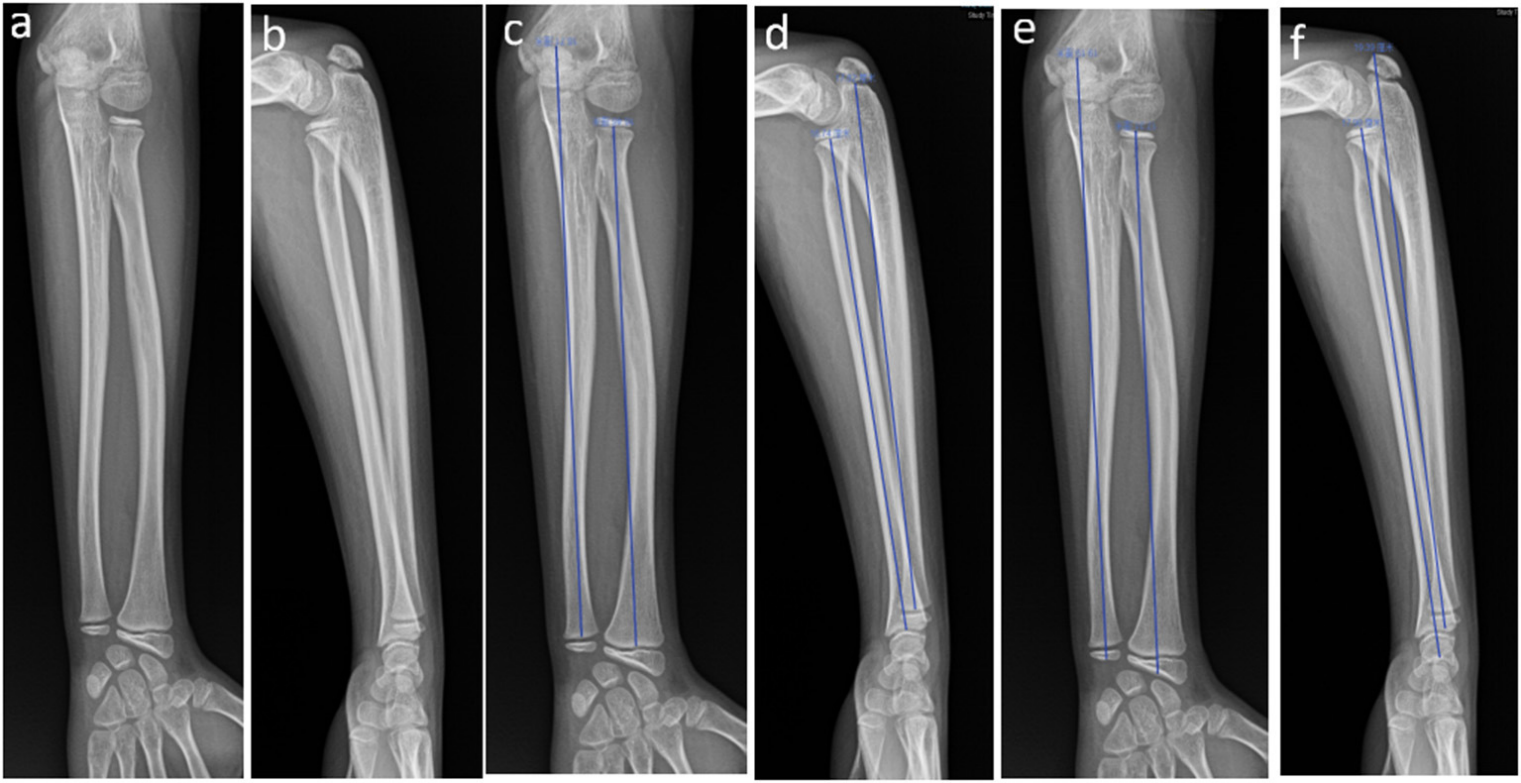
Anterior–posterior (AP) and lateral (LAT) radiographs of the left radius and ulna in a 10-year-old girl. **(a)** AP of the left forearm; **(b)** LAT of the left forearm; **(c)** AP showing measurement of the SLR and SLU; **(d)** LAT showing measurement of the SLR and SLU; **(e)** AP showing measurement of the TLR and TLU; **(f)** LAT showing measurement of the TLR and TLU.

Figure 1 is a graphical representation of the measurement of TLR, TLU, SLR, and SLU on AP and LAT radiographs.

## Results

### Data

A total of 326 subjects (192 boys and 134 girls, 173 left and 153 right) were enrolled in this study. Between 18 and 29 patients were enrolled in each age group ranging from 1 to 14 years old, 7 patients were in the 15 year-old group, and four in the 16 year-old group.

We assessed interobserver agreement using the intraclass correlation coefficient (ICC). Poor reliability was suggested for values between 0 and 0.20; fair reliability from 0.21 to 0.40; moderate reliability from 0.41 to 0.60; substantial or good reliability from 0.61 to 0.80, and almost perfect or very good reliability from 0.81 to 1.0 (24). The interobserver agreement was considered almost perfect, the ICC varied between 0.90 and 0.99 for all analyses.

### Age of appearance and close of radial epiphyseal plate

Due to the characteristics of radius and ulna development in children, the epiphyseal plate and the ossification center appeared and closed at both ends of the radius and ulna at different ages. In this study, the age of appearance of the epiphyseal plate and ossification center at the distal and proximal parts of the radius and ulna were different, but there were also objective rules. In the development of the radius, the earliest epiphyseal plate and ossification center occurred in the distal part of the radius at 1 year old both in boys (3/15 boys, 20%) and girls (9/11 girls, 82%), in the radial head at 3 years old both in boys (3/11, 27%) and girls (2/12, 17%). In the development of the ulna, the earliest epiphyseal plate and ossification center appeared in the distal part of the ulna at 7 years old in boys (1/15, 7%), at 6 years old in girls (2/7, 29%), and in the proximal part at 9 years old both in boys (1/11, 9%) and girls (11/14, 79%). Fusion of the ossification center of the ulnar olecranon began at 13 years old in boys (9/15, 60%) and at 12 years old in girls (4/4, 100%) (Tables 1, 2).

**Table 1:** Age (in years) of appearance and fusion of the forearm ossification centers for boys and girls.

	**Age of appearance of ossification center**				**Age of fusion of ossification center**
	Radial distal epiphysis	Radial head	Ulnar distal epiphysis	Ulnar olecranon	Ulnar olecranon
Boys	1y (3/15, 20%)	3y (3/11, 27%)	7y (1/15, 7%)	9y (1/11, 9%)	13y (9/15, 60%)
Girls	1y (9/11, 82%)	3y (2/12, 17%)	6y (2/7, 29%)	9y (11/14,79%)	12y (4/4, 100%)

**Table 2:** The length of the radius and ulna, and the radius: ulna ratio, at different ages.

**Age(N)**	**Total length (Mean**±**SD)**						**Short length (not including epiphysis) (Mean**±**SD)**					
	**AP radius**	**AP ulna**	**AP R/U**	**LAT radius**	**LAT ulna**	**LAT R/U**	**AP radius**	**AP ulna**	**AP R/U**	**LAT radius**	**LAT ulna**	**LAT R/U**
1Y (27)	Epiphysis not shown in X-Ray						8.44 ± 0.74	9.43 ± 0.79	0.8947 ± 0.1816	8.45 ± 0.68	9.45 ± 0.71	0.8936 ± 0.1705
Boys = 16							8.54 ± 0.78	9.46 ± 0.84	0.9031 ± 0.0144	8.54 ± 0.68	9.50 ± 0.70	0.8987 ± 0.0165
Girls = 11							8.29 ± 0.67	9.39 ± 0.76	0.8823 ± 0.0163	8.31 ± 0.68	9.37 ± 0.76	0.8862 ± 0.0156
2Y (29)							9.88 ± 0.50	11.05 ± 0.54	0.8941 ± 0.0194	9.96 ± 0.49	11.13 ± 0.56	0.8964 ± 0.0222
Boys = 7							9.96 ± 0.44	10.98 ± 0.36	0.9073 ± 0.0216	10.03 ± 0.47	11.04 ± 0.42	0.9083 ± 0.0226
Girls = 22							9.86 ± 0.52	11.08 ± 0.60	0.8899 ± 0.0171	9.95 ± 0.50	11.15 ± 0.61	0.8926 ± 0.0212
3Y (23)							11.32 ± 0.82	12.55 ± 0.87	0.9024 ± 0.1228	11.42 ± 0.84	12.62 ± 0.90	0.9049 ± 0.1327
Boys = 11							11.39 ± 0.67	12.60 ± 0.71	0.9042 ± 0.0111	11.50 ± 0.75	12.63 ± 0.73	0.9099 ± 0.0114
Girls = 12							11.26 ± 0.96	12.50 ± 1.02	0.9007 ± 0.0135	11.36 ± 0.94	12.62 ± 1.07	0.9003 ± 0.0137
4Y (21)							11.96 ± 0.70	13.27 ± 0.73	0.9014 ± 0.0176	12.10 ± 0.74	13.36 ± 0.75	0.9058 ± 0.0162
Boys = 10							12.22 ± 0.81	13.46 ± 0.91	0.9081 ± 0.0176	12.39 ± 0.83	13.52 ± 0.92	0.9168 ± 0.0143
Girls = 11							11.73 ± 0.51	13.10 ± 0.50	0.8953 ± 0.0161	11.84 ± 0.56	13.21 ± 0.56	0.8959 ± 0.0107
5Y (20)							13.25 ± 0.97	14.61 ± 0.94	0.9070 ± 0.0163	13.40 ± 0.99	14.70 ± 0.94	0.9111 ± 0.0193
Boys = 12							13.61 ± 0.72	14.91 ± 0.72	0.9125 ± 0.0123	13.76 ± 0.72	14.96 ± 0.72	0.9199 ± 0.0103
Girls = 8							12.73 ± 1.17	14.15 ± 1.14	0.8988 ± 0.0197	12.86 ± 1.18	14.32 ± 1.20	0.8980 ± 0.0240
6Y (25)							14.53 ± 1.28	15.97 ± 1.28	0.9092 ± 0.0160	14.71 ± 1.29	16.02 ± 1.33	0.9178 ± 0.0165
Boys = 18							14.96 ± 1.21	16.40 ± 1.20	0.9115 ± 0.0167	15.14 ± 1.23	16.46 ± 1.26	0.9198 ± 0.0175
Girls = 7							13.42 ± 0.63	14.86 ± 0.73	0.9034 ± 0.0134	13.60 ± 0.61	14.91 ± 0.72	0.9127 ± 0.0131
7Y (22)							15.33 ± 0.88	16.79 ± 0.88	0.9129 ± 0.0129	15.49 ± 0.92	16.82 ± 0.92	0.9211 ± 0.0132
Boys = 15							15.67 ± 0.75	17.09 ± 0.74	0.9166 ± 0.0108	15.83 ± 0.78	17.16 ± 0.72	0.9227 ± 0.0116
Girls = 7							14.61 ± 0.71	16.15 ± 0.87	0.9049 ± 0.0142	14.76 ± 0.78	16.09 ± 0.92	0.9178 ± 0.0166
8Y (19)							16.19 ± 1.22	17.80 ± 1.15	0.9086 ± 0.0188	16.40 ± 1.24	17.83 ± 1.21	0.9198 ± 0.0177
Boys = 11							16.70 ± 1.02	18.18 ± 1.15	0.9187 ± 0.0161	16.95 ± 1.00	18.26 ± 1.11	0.9281 ± 0.0157
Girls = 8							15.48 ± 1.17	17.29 ± 1.15	0.8947 ± 0.0126	15.66 ± 1.19	17.23 ± 1.13	0.9085 ± 0.0140
9Y (25)	18.59 ± 1.50	20.03 ± 1.65	0.9286 ± 0.0200	19.23 ± 1.55	19.93 ± 1.63	0.9648 ± 0.0169	17.79 ± 1.40	19.60 ± 1.64	0.9087 ± 0.0232	17.99 ± 1.47	19.33 ± 1.50	0.9302 ± 0.0201
Boys = 11	18.47 ± 1.20	19.69 ± 1.30	0.9383 ± 0.0147	19.14 ± 1.25	19.66 ± 1.33	0.9740 ± 0.0168	17.69 ± 1.09	19.25 ± 1.25	0.9193 ± 0.0178	17.91 ± 1.13	19.11 ± 1.26	0.9374 ± 0.0216
Girls = 14	18.68 ± 1.73	20.29 ± 1.89	0.9209 ± 0.0208	19.29 ± 1.79	20.15 ± 1.85	0.9575 ± 0.0137	17.87 ± 1.63	19.86 ± 1.90	0.9004 ± 0.0242	18.05 ± 1.73	19.51 ± 1.68	0.9245 ± 0.0176
10Y (24)	19.52 ± 2.23	20.55 ± 1.42	0.9508 ± 0.0947	19.78 ± 1.25	20.41 ± 1.45	0.9698 ± 0.0218	18.26 ± 1.16	20.16 ± 1.38	0.9063 ± 0.0.84	18.51 ± 1.17	19.88 ± 1.24	0.9313 ± 0.0181
Boys = 11	19.58 ± 1.17	20.84 ± 1.44	0.9404 ± 0.0201	20.29 ± 1.23	20.77 ± 1.37	0.9776 ± 0.0194	18.64 ± 1.14	20.44 ± 1.38	0.9124 ± 0.0167	18.91 ± 1.14	20.20 ± 1.12	0.9364 ± 0.0180
Girls = 13	19.47 ± 2.91	20.30 ± 1.41	0.9595 ± 0.1291	19.35 ± 1.14	20.12 ± 1.49	0.9631 ± 0.0222	17.93 ± 1.12	19.91 ± 1.38	0.9011 ± 0.0189	18.16 ± 1.12	19.60 ± 1.32	0.9270 ± 0.0177
11Y (21)	20.63 ± 1.55	22.45 ± 1.69	0.9192 ± 0.0187	21.47 ± 1.52	22.41 ± 1.64	0.9584 ± 0.0159	19.87 ± 1.48	21.74 ± 1.57	0.9114 ± 0.0220	20.04 ± 1.42	21.73 ± 1.37	0.9220 ± 0.0221
Boys = 10	21.42 ± 1.81	22.99 ± 2.17	0.9324 ± 0.0140	22.21 ± 1.76	23.01 ± 2.06	0.9662 ± 0.0116	20.53 ± 1.74	22.22 ± 1.93	0.9239 ± 0.0091	20.74 ± 1.60	22.15 ± 1.69	0.9366 ± 0.0176
Girls = 11	19.92 ± 0.80	21.96 ± 0.95	0.9072 ± 0.0138	20.79 ± 0.89	21.86 ± 0.92	0.9513 ± 0.0163	19.16 ± 0.83	21.30 ± 1.05	0.9001 ± 0.0244	19.40 ± 0.89	21.34 ± 0.93	0.9088 ± 0.0172
12Y (23)	21.43 ± 1.56	23.37 ± 1.82	0.9177 ± 0.0176	22.34 ± 1.66	23.34 ± 1.77	0.9579 ± 0.0234	20.58 ± 1.57	22.7 ± 1.64	0.9067 ± 0.0196	20.89 ± 1.52	22.79 ± 1.65	0.9173 ± 0.0230
Boys = 19	21.43 ± 1.54	23.31 ± 1.86	0.9203 ± 0.0176	22.44 ± 1.66	23.29 ± 1.85	0.9642 ± 0.0194	20.53 ± 1.56	22.60 ± 1.63	0.9083 ± 0.0209	20.90 ± 1.50	22.70 ± 1.70	0.9212 ± 0.0215
Girls = 4	21.43 ± 1.90	23.66 ± 1.80	0.9052 ± 0.0129	21.89 ± 1.83	23.56 ± 1.55	0.9283 ± 0.0185	20.84 ± 1.83	23.18 ± 1.82	0.8987 ± 0.0104	20.85 ± 1.86	23.18 ± 1.57	0.8987 ± 0.0234
13Y (18)	23.32 ± 1.66	25.28 ± 1.88	0.9230 ± 0.0143	24.25 ± 1.74	25.19 ± 1.76	0.9626 ± 0.0163	22.23 ± 1.67	24.05 ± 2.02	0.9251 ± 0.0218	22.58 ± 1.75	24.09 ± 1.92	0.9375 ± 0.0195
Boys = 14	23.09 ± 1.65	25.01 ± 1.87	0.9235 ± 0.0154	23.99 ± 1.70	24.91 ± 1.73	0.9632 ± 0.0167	21.96 ± 1.65	23.70 ± 1.93	0.9275 ± 0.0226	22.29 ± 1.71	23.76 ± 1.84	0.9383 ± 0.0213
Girls = 3	24.49 ± 1.44	26.61 ± 1.56	0.9206 ± 0.0072	25.54 ± 1.57	26.61 ± 1.30	0.9593 ± 0.0172	23.47 ± 1.40	25.69 ± 1.92	0.9142 ± 0.0167	23.91 ± 1.50	25.61 ± 1.81	0.9340 ± 0.0086
14Y (18)	22.73 ± 1.19	24.79 ± 1.22	0.9166 ± 0.0102	23.71 ± 1.14	24.86 ± 1.23	0.9538 ± 0.0080	Proximal ulna ossification fusion from 14 years old					
Boys = 16	22.77 ± 1.18	24.82 ± 1.20	0.9175 ± 0.0104	23.76 ± 1.08	14.91 ± 1.21	0.9542 ± 0.0077	
Girls = 2	22.35 ± 1.73	24.59 ± 1.82	0.9088 ± 0.0027	23.27 ± 2.00	24.47 ± 1.77	0.9505 ± 0.0128	
15Y (7)	24.54 ± 1.42	26.65 ± 1.65	0.9210 ± 0.0155	25.51 ± 1.46	26.42 ± 1.52	0.9656 ± 0.0070	
Boys = 6	25.03 ± 0.65	27.10 ± 1.08	0.9220 ± 0.0167	26.03 ± 0.56	26.93 ± 0.74	0.9665 ± 0.0071	
Girls = 1	21.63	23.64	0.9150	22.40	23.33	0.9651	
16Y (4)	23.38 ± 1.03	25.97 ± 0.79	0.9000 ± 0.0130	24.41 ± 0.95	25.75 ± 0.98	0.9481 ± 0.0124	
Boys = 4	23.38 ± 1.03	25.97 ± 0.79	0.9000 ± 0.0130	24.41 ± -.95	25.75 ± 0.98	0.9481 ± 0.0124	
Girls = 0	/	/	/	/	/	/	

### Characteristics of the length and ratio of radius and ulna

Since epiphyseal plates and ossification centers do not develop at the proximal part of the ulna until 9 years of age, measurement of the TLU and TLR began at 9 years old and continued up to 16 years old. The epiphyseal plate began to close in some children from 14 years old, so because the SLU and SLR needed to exclude the epiphyseal plate and the ossification center, this was measured from 1 year old up to 13 years old.

The lengths, and the ratio between the radius and ulna, differed according to age, as shown in Table 3.

**Table 3:** Ulnar length, radial length, age correlation coefficient and regression equation.

	**Pearson coefficient**				**Linear regression**			
**Length (CM)**	**AP**	**LAT**	**AP**	**LAT**	**AP**	**LAT**	**AP**	**LAT**
**Age (Y)**	**SL**	**SL**	**TL**	**TL**	**SL**	**SL**	**TL**	**TL**
Radial length & ulnar length (total)	0.997^**^	0.997^**^	0.933^**^	0.988^**^	UL = 0.396 + 1.075 RL^**^	UL = 0.707 + 1.043 RL^**^	UL = 1.139 + 1.028 RL^**^	UL = −0.415 + 1.060 RL^**^
Boys	0.997^**^	0.997^**^	0.988^**^	0.990^**^	UL = 0.338 + 1.072 RL^**^	UL = 0.599 + 1.044 RL^**^	UL = −1.717 + 1.161 RL^**^	UL = −1.252 + 1.093 RL^**^
Girls	0.997^**^	0.997^**^	0.809^**^	0.984^**^	UL = 0.272 + 1.094 RL^**^	UL = 0.682 + 1.052 RL^**^	UL = 5.918 + 0.785 RL^**^	UL = −0.268 + 1.061 RL^**^
Radial length & age	0.964^**^	0.965^**^	0.723^**^	0.783^**^	RL = 7.668 + 1.097 age^**^	RL = 7.692 + 1.120 age^**^	RL = 10.724 + 0.893 age^**^	RL = 10.380 + 0.987 age^**^
Boys	0.963^**^	0.965^**^	0.75^**^	0.766^**^	RL = 7.951 + 1.086 age^**^	RL = 7.962 + 1.112 age^**^	RL = 11.224 + 0.859 age^**^	RL = 11.419 + 0.916 age^**^
Girls	0.965^**^	0.964^**^	0.557^**^	0.690^**^	R = 7.459 + 1.089 age^**^	RL = 7.505 + 1.1.5 age^**^	RL = 10.673 + 0.882 age^**^	RL = 10.049 + 0.988 age^**^
Ulnar length and age	0.964^**^	0.965^**^	0.782^**^	0.789^**^	UL = 8.618 + 1.184 age^**^	UL = 8.698 + 1.171 age^**^	UL = 10.474 + 1.062 age^**^	UL = 10.342 + 1.067 age^**^
Boys	0.963^**^	0.964^**^	0.771^**^	0.779^**^	RL = 8.842 + 1.168 age^**^	UL = 8.894 + 1.163 age^**^	UL = 10.821 + 1.038 age^**^	UL = 10.893 + 1.028 age^**^
Girls	0.964^**^	0.965^**^	0.700^**^	0.703^**^	RL = 8.423 + 1.193 age^**^	UL = 8.552 + 1.167 age^**^	UL = 10.225 + 1.074 age^**^	UL = 10.005_1.085 age^**^
Radial/Ulnar length and age (R/U and age)	0.310^**^	0.518^**^	−0.183^*^	−0.195^*^	R/U = 0.895 + 0.002 age^**^	R/U = 0.894 + 0.003 age^**^	R/U = 0.972–0.004 age^**^	U/L = 0.982–0.002 age^**^
Boys	0.285^**^	0.488^**^	−0.493^**^	−0.400^**^	R/U = 0.904 + 0.001 age^**^	R/U = 0.903 + 0.003 age^**^	R/U = 0.981–0.005 age^**^	R/U = 1.007–0.003 age^**^
Girls	0.261^**^	0.521^**^	−0.110^**^	−0.193	R/U = 0.889 + 0.001 age^**^	R/U = 0.887 + 0.003^**^	R/u = 0.979–0.005 age^**^	R/U = 0.980–0.002 age^**^

*The radial and ulnar length showed a strong positive correlation with increasing age. The short length, total length of the radius and ulna measured by AP and LAT all showed a strong positive correlation with age (P <0.01), both in boys and girls (P <0.01). At the same time, the linear regression equation was calculated using the radius, ulna and age. Details are shown in this table. UL, ulnar length; RL, radial length; SL, short length; TL, total length*.

** *P <0.01,*

* *P <0.05*.

**Figure 2:**
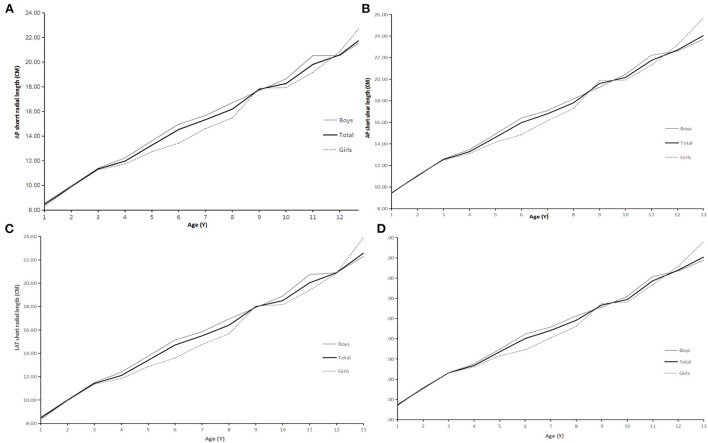
**(A)** AP short radial length and age. **(B)** AP short ulnar length and age. **(C)** LAT short radial length and age. **(D)** LAT short ulnar length and age.

**Figure 3:**
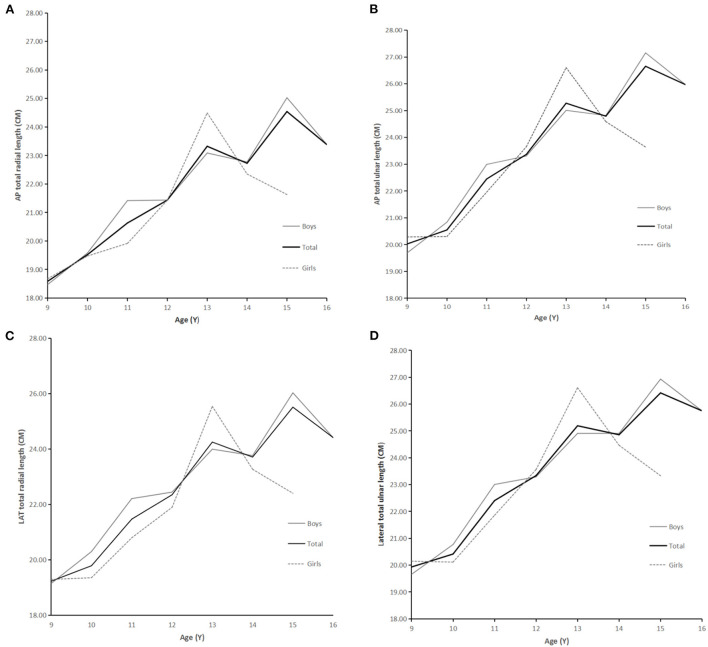
**(A)** AP total radial length and age. **(B)** AP total ulnar length and age. **(C)** LAT total radial length and age. **(D)** LAT total ulnar length and age.

The ulna and radius gradually become longer with age, as shown in Figures 2A–D, 3A–D.

The RLRU increased slightly with age, but remained stable and fluctuated only within a narrow range, whether measured by AP, LAT, short length, or total length. However, one-way analysis of variance (ANOVA) was conducted to evaluate the RLRU data in each age group, and the *P* values of homogeneity of variance test were all >0.05, indicating homogeneity of variance. On this basis, the LSD method was used to compare the RLRU of different age-groups under the four different measurements.

**Figure 4:**
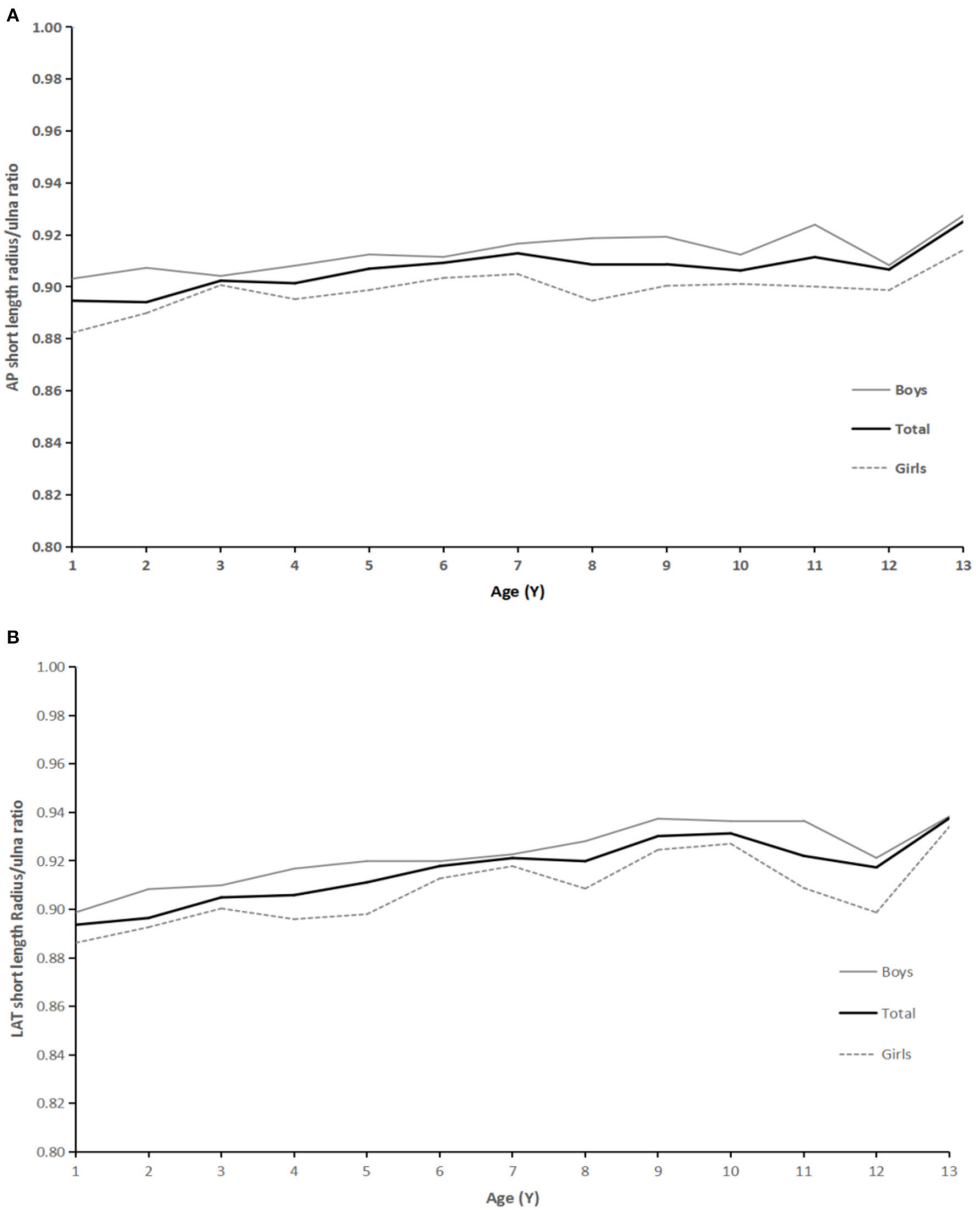
**(A)** AP radiographs: relationship between age and ratio of radius:ulna short length. **(B)** LAT: relationship between age and ratio of radius: ulna short length.

**Figure 5:**
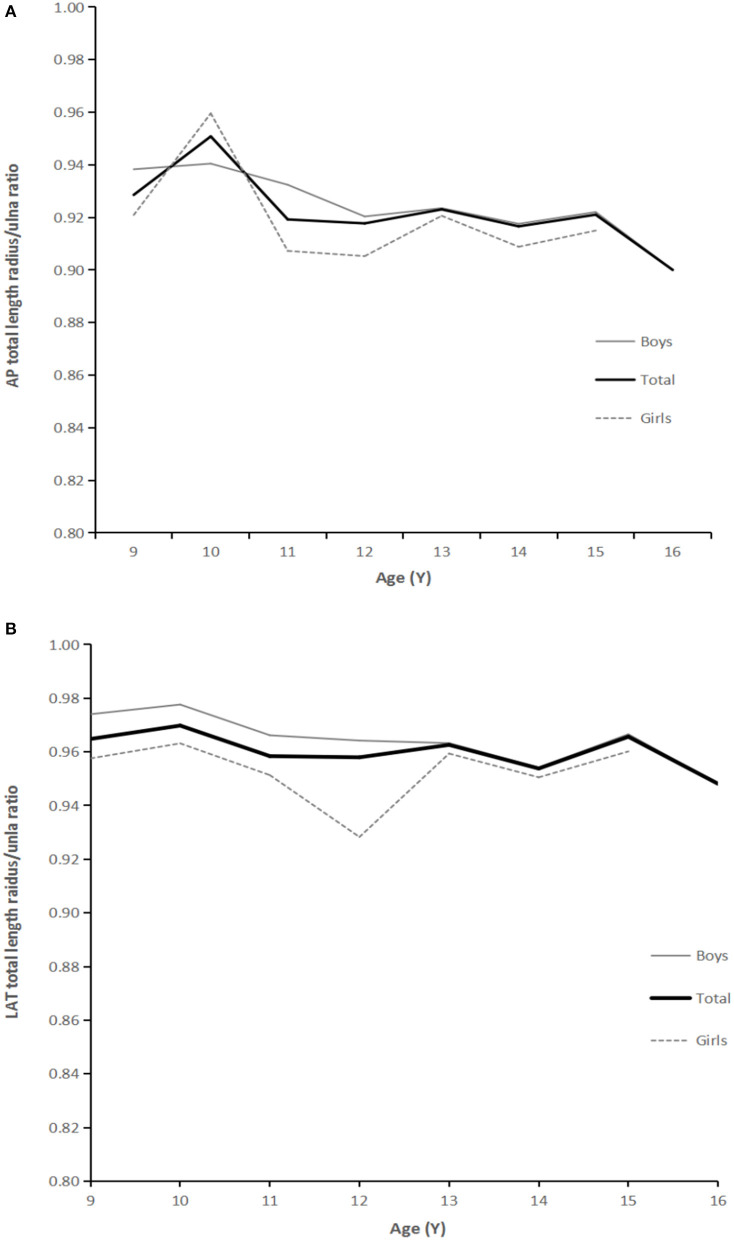
**(A)** AP radiographs: relationship between age and ratio of radius:ulna total length. **(B)** LAT: relationship between age and ratio of radius: ulna total length.

For short length measurement results (1–13 years old), there were significant differences between different ages (*P* < 0.01). Specifically, with the increase in age (1–13 years old), the radius/ulna ratio (short length) showed a gradually increasing but significant trend. From 1 to 4 years old, the radius/ulna ratio increased with age and became relatively stable after 5 years old. However, in both AP and LAT, short length ratios were from 0.89 to 0.94 (Figures 4A,B). There was no significant difference in the ratio of radius/ulna (total length) in AP and LAT in groups from 9 to13 years old (AP, *P* = 0.111; LAT, *P* = 0.21). Even with the slightly higher at 10 years old, the ratio remained stable. In the case of total length measured on AP radiographs, it was restricted to between 0.91 to 0.95, and in the cases of total length measured on LAT radiographs, it was restricted from 0.95 to 0.97 (Figures 5A,B).

## Discussion

There has been a lot of research on the lower limbs (25, 26), however there is a lack of research into the natural length and angle of the upper limbs. Upper limb diseases and disorders such as congenital anomalies, tumors and endocrine metabolic abnormalities can cause deformities of the radius and ulna. Currently, these deformities may be treated by lengthening the radius and/or ulna using the extension technique of osteotomy with external fixator, realizing the aim of correcting the deformity and thus restoring limb appearance and function (4–10).

For clinical surgical treatment, there is a lack of objective and accurate measurement data and analysis such as X-rays. The appearance and function of the forearm are compensated by the wrist joint, elbow joint, and shoulder joint. Thus, the need for the length and ratio of the ulna and radius to be corrected is not so urgent (16), and research into the length and ratio of the ulna and radius is lacking. In addition, ethical issues make it impossible to measure the lengths of the normal radius and ulna using X-rays. This study used normal radial and ulnar X-rays taken from children treated for trauma in the emergency department of our hospital, excluding fractures and other deformities of the forearm, thus the study was IRB approved, avoiding ethical problems.

In this study, the proximal and distal parts of the radius and ulna with or without epiphyseal plates and ossification centers were used for measurement; however a mixture of data with or without inclusion of the epiphyseal plate caused confusion, preventing us from making the right analysis and judgment. Therefore, we suggest the concepts of “short length” which excludes the proximal and distal epiphyseal plates of the radius and ulna, and “total length” which includes both the proximal and distal epiphyseal plates of the radius and ulna. Any length measurements which consisted of one end with the epiphyseal plate and the other end without the epiphyseal plate were not included in the statistical analysis.

The second aspect that needs to be addressed is measurement criteria. Since there are concepts such as anatomical axis and mechanical axis which are used in lower limb measurement, most measurements adopt the connection between the midpoint of the joints on both sides or the midpoint of the shaft (27), so ulna and radius measurement also refers to the method of connecting the midpoint at both ends of the ulna and radius. However, it is difficult to determine the midpoint at the proximal end of the ulna when the “total length” is actually measured on AP and LAT radiographs, so the most prominent point at the proximal end of the ulna is used instead of the midpoint to improve the accuracy and repeatability of the measurements.

The epiphyses of the radius and ulna, as well as secondary ossification centers, appeared on X-rays at different times in different age groups. For example, the earliest appearance of epiphyseal plates and ossification centers at the distal end of the radius was at 1 year old, and at the proximal end was 3 years old. The earliest appearance of epiphyseal plates and ossification centers at the distal end of the ulna was 6 years old in girls and 7 years in boys, while at the proximal end it was 9 years old in both boys and girls. These observations were similar to others reported in the literature (24, 28–32).

The lengths of both the radius and ulna increase with age, but there is a strong correlation between length and age, and the corresponding linear regression equation can be calculated. These are important references for predicting the development of radial and ulnar length. Moreover, although the ratio of the length of the radius to that of the ulna increases slightly with age, it is basically maintained within a relatively stable and narrow range, especially in older adolescents. Thus, the ratio of the length of the radius to the ulna is basically kept constant and is not affected by age. These rules suggest that there is a corresponding ratio between the upper limbs, especially the ulna and radius of the forearm, and that this is closely related to age. Only by maintaining the corresponding relationship of these basic features can the structure remain a stable and functional moving complex.

Our study has revealed that there are rules regarding the length of the forearm, there may also be some rules regarding the angles of the forearm. It is possible to introduce the concepts of anatomical and mechanical axes as in lower extremity measurement, joint walking direction, different plane angle formation and bone osteotomy at the center of rotation of angulation (CORA). Rules regarding the angles of the forearm may help us to further implement accurate prediction and treatment for the correction of forearm and upper limb deformity numerically, and thus achieve the best treatment effect. If these characteristics are similar to those of the lower extremities, then for humans who walk on two lower limbs and have their upper limbs liberated to engage in complex activities, the correction of forearm deformity will be supported by more data. It is possible to achieve the ideal recovery state, the best correction of appearance deformity and functional recovery.

Because of the limited number of cases, these data are still speculative. With further research into the relationship between the length and ratio of the deformed radius and ulna, and the development and verification of upper limb deformity correction surgery, these questions may be answered. These will further promote progress in the treatment of upper limb deformity.

## Data availability statement

The raw data supporting the conclusions of this article will be made available by the authors, without undue reservation.

## Ethics statement

The study was conducted in accordance with the Declaration of Helsinki (as revised in 2013). This study was retrospective, it was approved by Ethics Committee of Children's Hospital of Fudan University and individual consent for this retrospective analysis was waived.

## Author contributions

CW: study design, performed measurements, cases collection, statistical analysis, and manuscript preparation. DW: study design, performed measurements, and manuscript preparation. YM: performed measurements, statistical analysis, and cases collection. ZZ: cases collection and performed measurements. BN: study design, performed measurements, manuscript preparation, supervision, validation, responsible for literature search, study design, and finalized the manuscript. All authors contributed to the article and approved the submitted version.

## Funding

This work was supported by Science and Technology Commission of Shanghai Municipality (CN) (No. 20Y11912900), Medical Innovation Research Project of Science and Technology Innovation Action Plan of Shanghai Science and Technology Committee 2020, and Key Development Program of Children's Hospital of Fudan University (No. EK2022ZX03).

## Conflict of interest

The authors declare that the research was conducted in the absence of any commercial or financial relationships that could be construed as a potential conflict of interest.

## Publisher's note

All claims expressed in this article are solely those of the authors and do not necessarily represent those of their affiliated organizations, or those of the publisher, the editors and the reviewers. Any product that may be evaluated in this article, or claim that may be made by its manufacturer, is not guaranteed or endorsed by the publisher.
